# A dataset for document level Chinese financial event extraction

**DOI:** 10.1038/s41597-025-05083-9

**Published:** 2025-05-10

**Authors:** Yubo Chen, Tong Zhou, Sirui Li, Jun Zhao

**Affiliations:** 1 The Key Laboratory of Cognition and Decision Intelligence for Complex Systems, Institute of Automation, Chinese Academy of Sciences, Beijing, China; 2 School of Artificial Intelligence, University of Chinese Academy of Sciences, Beijing, 101408 China; 3 University of California Berkeley, Berkeley, CA USA

**Keywords:** Genetic databases, Events

## Abstract

Financial event modeling is fundamental to financial investment decisions and risk management, crucial for the stability and growth of financial institutions, and helps ensure the stability and quality of people’s lives. Utilizing state-of-the-art natural language processing techniques for automated financial event extraction addresses the inefficiencies and high costs associated with traditional event identification and modeling, which rely heavily on financial domain experts. However, existing datasets fail to tackle the issues with long documents in practical situations. To address this, we first propose DocFEE, a large-scale **Doc**ument-level Chinese **F**inancial **E**vent **E**xtraction dataset. It reflects the length of announcement documents and the long-distance dependencies of event arguments in real-world scenarios.

## Background & Summary

**Fig. 1:**
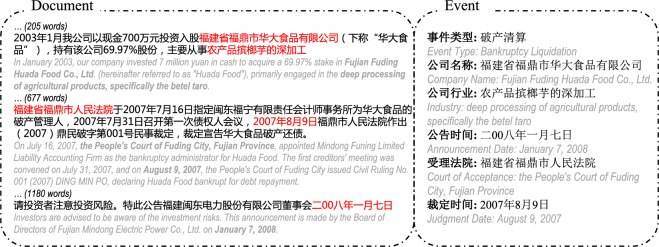
An example of financial document-level event extraction.

Financial events modeling can help the financial practitioner with investment decisions and risk management. These are critical not only for ensuring the stability and growth of financial institutions^1,2^ but also for safeguarding the overall security and sustainable development of both financial markets and global economies^3,4^. For example, the occurrence of an equity freeze event can severely impact a company’s operations and shareholder value, underscoring the urgency for timely and effective decision-making by stakeholders. Financial event extraction is the cornerstone in financial events modeling, which aims to identify and extract pertinent information about financial events from nature language text, including the event type, participants, time, location, and other valuable arguments^5–9^. Fig. 1 shows an example of this task’s input and output. Effective extraction of financial events can be utilized for monitoring and identifying risks, aiding in the quantitative assessment of risks, supporting decision-making, and, most importantly, accumulating data to construct knowledge graphs, which form the foundational infrastructure for subsequent event modeling and prediction .

Traditional financial event extraction is manually conducted by humans^10,11^. The financial expert summarizes and extracts various predefined events by comprehending and analyzing financial documents^12^. This process is highly dependent on their financial knowledge and industry experience, which makes it costly, inefficient, and susceptible to subjective decision-making biases^13,14^. In contrast, automated approaches facilitated by machine learning and deep learning algorithms offer promising solutions to mitigate these drawbacks^15,16^. Recent natural language processing advancements have enabled automated extraction and modeling of financial-related events from unstructured texts^17–24^. Such capabilities form a foundational basis for proactive risk alerting and trend forecasting^25,26^. However, existing datasets for document-level event extraction in the financial domain fail to reflect real-world challenges concerning long-context dependency.

The existing dataset does not take into account real-world length public announcements for the financial event extraction task^7,15,27,28^. Financial domain texts such as company announcements, research reports, annual reports, and legal documents are lengthy and often scatter event arguments across multiple sentences. We conduct a statistical analysis of actual financial event announcements, revealing that the average length exceeds 2,000 Chinese characters, with the longest announcements reaching hundreds of thousands. Further, as demonstrated in Fig. 1, accurately representing these events necessitates long-distance semantic modeling. Existing datasets, however, inadequately capture the span and intricacy of these arguments in real-world scenarios.

To address this challenge, we introduce DocFEE, a novel dataset, and present HAC-Ann, its corresponding hierarchical annotation pipeline. We first introduce DocFEE, a large-scale dataset tailored for **Doc**ument-level Chinese **F**inancial **E**vent **E**xtraction, focusing specifically on announcements from listed companies in Table 1 the Chinese stock market. The dataset encompasses nine event categories and identifies 38 event arguments. Detailed statistical information is shown in Table 2. Notably, it doubles the document length and event arguments span compared to existing datasets, enhancing its realism and challenging the extraction task. The DocFEE dataset not only aids in enhancing the effective identification, quantification, and modeling of financial events but also contributes to the development of long-text understanding in the field of natural language processing through its real-world, lengthy announcement documents.

**Table 1:** Comparison of the cost and effectiveness of different annotation methods.

Method	Price/1k sample	Documents of Interest	
		Precision	Recall
Rule	0	56.10%	100.00%
Qwen1.5-14B-Chat	0.05	75.00%	78.26%
Qwen1.5-32B-Chat	0.09	80.36%	97.83%
gpt-3.5-turbo-0125	5	79.59%	84.78%
gpt-4o	50	85.37%	76.09%
human	500	100.00%	100.00%

The costs we present are in USD for the annotation of every 1000 data entries. For open-source models, we calculate the electricity consumption rate through local deployment on the A100 server. Commercial API models are calculated based on the average request cost. Documents of interest refer to documents that contain at least one target financial event. This metric is used to demonstrate the efficiency and effectiveness of the model in filtering out irrelevant documents.

**Table 2:** Statistical information of DocFEE.

Key	Value
Dataset Name	DocFEE
Size	19,044
Event Type	9
Argument Type	38
Event Range	960.06
Event Count	1.86
Document Length	2277.25

The calculation method for the Event Range is the average distance from the first character of the first argument that appears in the document for each event to the last character of the last argument.

We utilize a low-cost and efficient **H**uman-**A**I **C**ollaborative training data **Ann**otation framework, HAC-Ann, for DocFEE’s construction. HAC-Ann leverages large language models’ powerful semantic understanding capabilities to achieve large-scale, low-cost, and efficient annotation based on task comprehension. This framework combines meticulously designed mechanisms for judging and filtering high-quality annotation results, automatically retrying or discarding potential low-quality annotations. Human annotation experts play a dual role: guiding large language models through initial dataset annotation to facilitate in-context learning of tasks and iteratively optimizing examples and instructions tailored to address the model’s annotation deficiencies. This iterative process aims to achieve iterative improvements with human feedback loops.

We further validated the effectiveness of the dataset through performance improvements achieved by different baselines trained on datasets of varying scales. The results indicate that training on a larger DocFEE data set can benefit various event extraction paradigms. Additionally, we demonstrate the credibility of the data annotation process method through manual verification. The contributions of this paper are summarized as follows:

- We present a large-scale document-level Chinese financial announcement event extraction dataset, DocFEE, characterized by its extended document lengths and event complexity. DocFEE covers nine types of events and 38 categories of event arguments, the dataset comprises 19,044 announcement documents with an average length of 2,277 Chinese characters and an average event argument span exceeding 960 Chinese characters.
- Manual evaluation of the DocFEE proves the authority of the sim-automatic constructed dataset, and the improvement of various baselines with the increase in training dataset validates the effectiveness of the DocFEE in financial event extraction.
- The dataset DocFEE helps optimize and enhance financial events modeling and promotes the advancement of long-text NLP research.

## Methods

### Task Paradigm

We focus on document-level financial event extraction from company announcements. This task is formulated as reading a long-form document with plain text and getting specified target events and their corresponding event arguments. As depicted in Fig.1, the input consists of a financial announcement comprising thousands of Chinese characters. This task aims to identify all events related to the financial domain and their corresponding arguments within the document. The ideal output would involve all structured event information in the document. In this instance, the announcement describes a bankruptcy liquidation event. The structural event information first points out the type, which in this case is bankruptcy liquidation, followed by the extraction of arguments associated with this event type. These arguments include the company name, industry, announcement date, and judgment date. The values of these arguments are to be extracted directly from the original text. For example, the judgment date is specified as August 9, 2007. Our end-to-end extraction process emphasizes key event arguments rather than event trigger words. This paradigm considers the implicit expression of the target event across multiple sentences, which can hardly find an appropriate trigger word. This adjustment ensures the generalizability and applicability of the event and corresponding arguments.

### Event Schema

The definition of event types in the DocFEE dataset is summarized by industry experts based on the Guidelines for Information Disclosure of Listed Companies (http://www.csrc.gov.cn/csrc_en/c102030/c1371072/content.shtml), covering the financial events with the highest impact. Specifically, the DocFEE dataset we have constructed covers nine types of financial events:

1. Bankruptcy Liquidation: This type of event refers to a company’s assets being sold off to pay its debts. This event signifies a company’s legal termination through asset liquidation to settle debts, often triggered by insolvency or court orders. It requires immediate disclosure under China’s disclosure rules due to its irreversible impact on shareholder equity and market stability. Regulatory authorities closely monitor such proceedings to ensure creditor hierarchies and investor rights compliance.
2. Major Safety Incident: This type of event involves significant accidents or events that compromise safety within the company, potentially causing financial and reputational damage. These involve catastrophic accidents (e.g., industrial disasters or environmental breaches) causing fatalities, operational shutdowns, or regulatory penalties. Mandated disclosures under regulations must quantify financial losses and reputational risks, as these incidents often prompt lawsuits, government investigations, and long-term operational restrictions.
3. Shareholder Reduction: This event occurs when a significant shareholder sells off a portion of their shares, which may affect the company’s stock price and investor confidence. Regulators require prompt disclosure to prevent insider trading risks, with regulators scrutinizing transactions for compliance with lock-up periods and market manipulation rules.
4. Equity Pledge: This event is when shareholders use their equity shares as collateral to secure loans. Shareholders’ use of equity as loan collateral introduces systemic risks, as defaults could trigger forced share disposals and market volatility. Disclosure obligations under regulations mandate reporting pledge ratios and lender details, enabling regulators to assess leveraged exposure and enforce margin call protocols.
5. Shareholder Increase: This event takes place when a significant shareholder acquires more shares, potentially affecting the company’s control and stock price.
6. Equity Freeze: This type of event happens when a court or regulatory authority restricts the sale or transfer of a company’s shares. Court-ordered restrictions on share transfers typically arise from litigation, debt recovery disputes, or regulatory sanctions.
7. Senior Executive Death: The sudden demise of key executives disrupts strategic continuity and may expose governance vulnerabilities. Companies must disclose succession plans and operational contingencies, as leadership vacuums risk contractual defaults and investor withdrawal.
8. Major Asset Loss: Significant asset impairment from natural disasters, fraud, or market collapses directly impacts balance sheets and credit ratings. Regulations requires detailed loss quantification and recovery strategies, with auditors verifying insurance coverage and impairment calculations under risk-oriented audit principles.
9. Major External Compensation: This event occurs when a company has to make substantial payments to external parties. Large payouts from lawsuits, fines, or regulatory settlements materially affect cash reserves and profitability forecasts. Disclosures must contextualize payments within ongoing legal exposures and compliance reforms.

For each type of event, we design corresponding event arguments. The detailed information is shown in Table 3. These events are crucial for financial modeling. The associated event arguments provide essential information that captures the complete picture of each event.

**Table 3:** Event types and corresponding event arguments.

Event Type	Arguments
Bankruptcy Liquidation	Company Name, Industry, Announcement Date, Court of Acceptance, Judgment Date
Major Safety Incident	Number of Casualties, Company Name, Announcement Date, Other Impacts, Loss Amount
Shareholder Reduction	Reduction Start Date, Shareholder, Reduction Amount
Equity Pledge	Receiver, Pledge Start Date, Pledgor, Pledge End Date, Pledge Amount
Shareholder Increase	Increase Start Date, Shareholder, Increase Amount
Equity Freeze	Freeze Start Date, Freeze End Date, Freeze Amount, Frozen Shareholder
Senior Executive Death	Company Name, Death/Missing Date, Age at Death, Executive, Position
Major Asset Loss	Company Name, Announcement Date, Other Losses, Loss Amount
Major External Compensation	Company Name, Announcement Date, Compensation Recipient, Compensation Amount

### Announcement Collection and Processing

We collect announcements from Chinese listed companies from January 2020 to April 2024 from EastMoney (https://data.eastmoney.com/notices/). This website records all the announcements released daily by listed companies in China, including titles, contents, and coarse-grained announcement-type tags. The pre-screen process is based on the tags provided by the website for each announcement. We exclude content that does not contain interest events with tags such as *Investor Relations* and *Amendment of Articles of Association*. The plain text is extracted from the PDF documents.

### Annotation Process

The objective of the annotation process is to identify financial announcement documents that contain the target events. All mentioned event types should be labeled for each document, and the corresponding event arguments’ segment positions within the document should be pinpointed.

We utilize an efficient Human-AI collaborative document-level event extraction annotation workflow that capitalizes on the generalization, cost-effectiveness, and efficiency of large language models and is complemented by human experts’ task comprehension and analytical expertise. The annotation process considers various annotation agents, including error-prone low-cost open-source large language models, capable commercial API endpoints, and the most expensive golden standard human annotators. HAC-Ann aims to trade-off between the cost and overall annotation quality, enabling this procedure to be conveniently deployed in labeling constantly emerging documents with target events.

The annotation process is divided into five recurrent steps: initial set annotation, rule-based filtering of irrelevant documents, model-based filtering of irrelevant documents, super-generation annotation, and human-in-the-loop optimization. Initially, a small subset must be manually annotated to initiate the entire annotation process, providing a foundation for subsequent AI learning and annotation performance optimization. Subsequently, due to the large volume of irrelevant documents that do not contain target events, a two-stage filtering using rules and models is necessary to ensure efficiency while minimizing false positives. The annotation results should be obtained through a super-generative annotation method based on LLM with an automatic verification mechanism, gradually gathering high-quality annotation outcomes. Finally, humans adjust the in-context learning examples and instruction requirements of the large language models based on the automatic evaluation results of the annotation outcomes, continuously enhancing the overall process’s effectiveness. We provide a detailed description of these steps below.

#### Initial Set Human Annotation

This initial set of human annotations aims to demonstrate the labeling guidelines for advanced large language models, facilitating more efficient and cost-effective large-scale annotation. It also serves as the foundation for subsequent automatic evaluation of annotation effectiveness, model selection, and recurrent optimization.

During the manual annotation process, due to the lengthy and challenging nature of financial documents, human annotators may make errors or omissions. Furthermore, differing interpretations of annotation standards among individuals may lead to subjective annotation results. We employ a consensus mechanism to guide the human annotation process in mitigating this issue.

Specifically, we initially engaged in discussions with senior practitioners who are experts in the financial domain to formulate and refine annotation guidelines and standard examples, with 15 instances for each event type. Subsequently, we recruited annotators from professionals in the securities industry as candidates. We train all candidates, equipping them with annotation guidelines, schema definitions, and five case examples per event type for learning purposes. Following the training, each candidate conducts a trial with an annotation of 10 data instances per event type and experiences a schema definition recitation test. Qualified annotators are required to demonstrate complete consistency with expert annotations during the trial phase and exhibit a thorough understanding of event types and their corresponding arguments. Ultimately, we selected 10 annotators.

To ensure the authority of the annotation results, each data instance is independently annotated by two different annotators. If the annotations are entirely consistent, the data instance is automatically included in the dataset. In cases of inconsistency, a senior expert with profound knowledge of financial event modeling reviews the two annotations and makes necessary revisions. The finalized annotation results were then communicated to the two annotators, accompanied by essential explanations. This approach maximizes the quality advantages of expert annotations while avoiding excessive costs.

Although human-annotated results are of high quality, they are costly and time-consuming. To balance the coverage of annotated data and costs, each annotator was responsible for annotating 800 valid data instances, resulting in a total of 4,000 annotated instances across all nine event types while also considering the balance and diversity of event categories.

#### Filtering Irrelevant Documents by Rules

This step aims to massively and efficiently filter out announcements that do not contain events associated with target scope since most corporate announcements do not involve the specified financial target events, thereby reducing unnecessary annotation costs in the following annotation steps. We propose a rule-based approach that is efficient and cost-effective; meanwhile, we have validated its effectiveness through evaluation, achieving a recall rate of 100%.

We formulate heuristic rules for retrieving documents containing the specified events based on keyword matching. The rules imply that if an announcement contains any single keyword from the set associated with a particular event type, we consider that the announcement may be expressing that event. We manually refine keyword sets based on human experts’ annotated dataset. The optimization objective of those rules is to achieve a 100% recall rate for the event type, with precisions at least 50%. The first row of Table 1 shows the hit situation of the heuristic rule set for documents containing the target event (documents of interest). Our rules achieve a precision rate of 56.10% while ensuring a recall rate of 100%.

#### Further Filtering by Model

Considering the low precision of documents retrieved through heuristic keyword rules, additional filtration is essential to refine the documents of interest. The objective of this step is to reduce the amount of manual labor needed for thorough annotation.

We propose a pre-labeling method based on large language models to filter out irrelevant documents. Specifically, we prompt LLMs to pre-labeling target events and arguments corresponding to the document and filter empty results. The prompt employs detailed instruction alongside diversified demonstrations, capitalizing on the LLM’s abilities in instruction following and in-context learning. With this elaborate prompt, LLM is able to acquire an understanding of the annotation guidelines, requirements, and output format. Notably, the demonstrations curated for LLM encompass documents devoid of any target events, which results in the direct generation of an empty list. Our focus at this stage is solely on the document of interest, with the goal that the LLM should not yield an empty list if it encompasses at least one of the nine events and, conversely, should produce an empty list to filter the document when no events are present.

We compute various annotation agents’ recall and precision rates for annotating non-empty lists, shown in the middle columns in Table 1. These results were derived from evaluating various models on subsets of the initial human-labeled dataset. We selected an open-source Qwen1.5-32B-Chat^29^ model to pre-label all data retrieved by keyword-based rules and discard documents lacking any events. Given that this model can attain a recall rate of 97.83% in documents of interest, the process incurs an acceptable data loss rate of less than 0.68% of all crawled announcements. This two-tiered filtering procedure can curtail the ultimate annotation expenses by 86.1%.

#### Super-Generative Annotation

Manual annotation is costly and inefficient. We utilize large language models for automatic annotation to achieve high efficiency and reduced costs. However, the response of the LLM is unstable, which means the same question posed multiple times may yield entirely different answers, which may include a mix of correct and incorrect ones. Leveraging this characteristic, we propose an auto-refine super-generation strategy to get high-quality annotation data.

We utilize heuristic rules to automatically filter out responses with evident annotation errors, and resort to an automatic consensus judgment mechanism to derive stable annotations from large language models, thereby preserving the potential for high-quality annotation results.

Heuristic Rules: The generated output from LLM may not conform to predefined requirements, including non-JSON format, non-predefined event types and arguments, argument values not derived from the span in the document, and event types outside the set of keyword rules results. These heuristic rules can automatically filter out low-quality annotations. If the current generation result is filtered out, the LLM must regenerate until the result meets the requirements of the heuristic rules.

Consensus Filtering: To mitigate the negative impact of occasional outlier responses from LLM, we initially let the LLM generate annotation results twice. If the two results are identical, they are considered stable predictive outcomes, which are more likely to be credible. If the results differ, the model continues to generate until a pair of identical results is obtained. This approach helps to retain more stable and higher-confidence annotation outcomes.

**Fig. 2:**
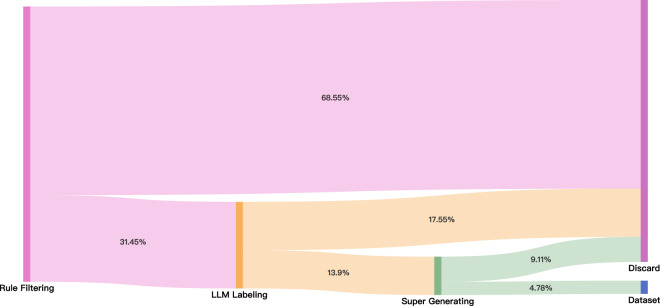
The filtering stream throughout the entire annotation process of the HAC-Ann pipeline.

As illustrated in Table 6, combining these two approaches into the auto-refine super-generation strategy exhibits enhanced data usability regarding argument F1 scores compared to single-pass annotations with the same model. Due to the model struggling to achieve annotation results that meet heuristic rules or produce stable outputs in some documents, we preset a maximum number of retries to keep the overall budget controllable. The data is discarded if the model fails to produce a stable output that meets the requirements after exceeding the retry limit. We utilize GPT-3.5-turbo (https://platform.openai.com/docs/models/gpt-3-5) with the auto-refine super-generation strategy to obtain the final automatic annotation dataset. The scale retention diagram of the data annotation process is depicted in Fig. 2.

#### Human-in-the-Loop Iterative Optimization

**Fig. 3:**
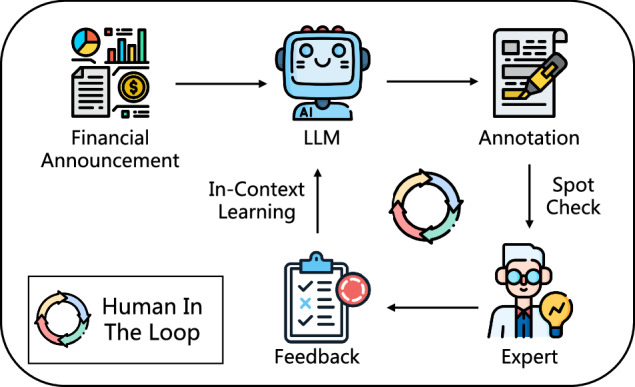
The human-in-the-loop iterative optimization process.

This step shown in Fig. 3, aims to enhance the quality of model annotations further. Although large language models possess powerful in-context learning capabilities, their proficiency in annotation tasks is significantly influenced by the clarity of instructions and the organization of few-shot examples. We can improve the instructions and the composition of examples by manually reviewing the errors in the current prompt’s annotation results.

We sample a subset of the dataset labeled by GPT-3.5-turbo with auto-refine super-generation and call for human annotators for further validation. By manually comparing human and GPT-3.5-turbo annotations, we identify event types where GPT-3.5-turbo underperforms and optimize the prompt with corresponding gold examples. Furthermore, by summarizing the types of errors, it is possible to emphasize such issues in the instructions. For instance, if it is observed that the content of event arguments generated freely by large language models contains a significant amount that is not from the original text segments, it is necessary to specify in the instructions, “Ensure that the extracted event argument content must be verbatim from the original text.” This iterative process improves annotation outcomes.

### Statistical Information

#### Overall Statistic

The DocFEE dataset comprises a substantial collection of 19,044 entries, encapsulating nine distinct event types and thirty-eight argument types. This extensive dataset has an event range calculated as the average distance between the initial argument’s first character and the final argument’s last character within each document, amounting to 960.06. On average, each document contains 1.86 events and has a length of 2277.25 characters. The event type definition encompasses significant events related to financial event modeling. Each event type is associated with specific arguments. For example, a Bankruptcy Liquidation event typically includes the company name, industry, announcement date, court of acceptance, and judgment date. These detailed and structured definitions facilitate comprehensive analysis and understanding of various financial events and their specific attributes.

#### Document-wise Statistic

As depicted in Table 4, regarding labeling methodology, DocFEE is the first to be constructed through a collaborative annotation approach (HAC-Ann) involving humans and LLMs. Previous datasets were annotated either through distant supervision or entirely reliant on manual labor, which is challenging to expand at a low cost and with high efficiency. The average document length of our dataset exceeds all previous datasets by over double times, presenting the challenge of dealing with these lengthy documents. The average number of events in our dataset is also the highest, making the scenario for event extraction more realistic and challenging. Besides, the average span range of event arguments within documents is nearly twice that of the DocFEE dataset, indicating that this dataset demonstrates difficulty modeling long-distance semantic dependencies.

**Table 4:** A comparative analysis of average statistical information for individual data entries across different datasets.

Dataset	Labeling Method	Event Range	Event Count	Document Length
DCFEE^7^	Distance	407.46	1.33	1109.87
ChFinAnn^15^	Distance	517.53	1.49	911.59
FEED^27^	Distance	515.59	1.48	907.89
DuEE-Fin^28^	Manual	252.92	1.35	497.30
DocFEE (Ours)	HAC-Ann	960.06	1.86	2277.25

**Fig. 4:**
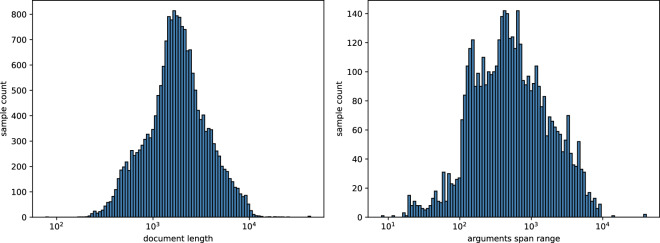
Left side: Frequency distribution of document lengths. Right side: Frequency distribution of event argument spans.

Figure 4 shows the frequency distribution of document lengths and the distribution of event argument spans across the corresponding document. The majority of document lengths fall between 1000 and 10,000 characters, while the spans of event arguments are mostly longer than 100 characters.

#### Event-wise Statistic

**Fig. 5:**
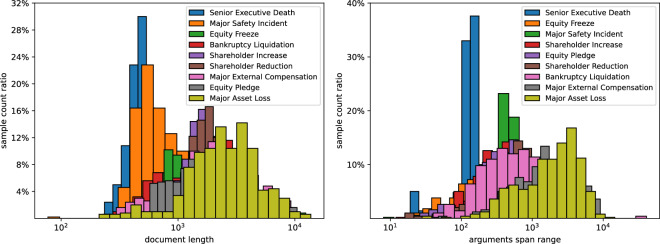
Left side: Proportional frequency distribution of document lengths corresponding to different event types. Right side: Proportional frequency distribution of event argument spans for different event types. The event types in the legend are ranked according to the mean of the horizontal axis, with events listed near the front having a smaller overall mean for the horizontal axis.

In conjunction with Fig. 5, it illustrates the proportional frequency distribution of document lengths and event argument spans corresponding to different event types. It is discernible that serious financial events, such as Senior Executive Deaths, Major Safety Incidents, and Equity Freezes, are typically documented in shorter announcements. Conversely, announcements about Major Asset Loss are longer and exhibit greater spans in event argument, because these events may appear in lengthy annual reports.

**Fig. 6:**
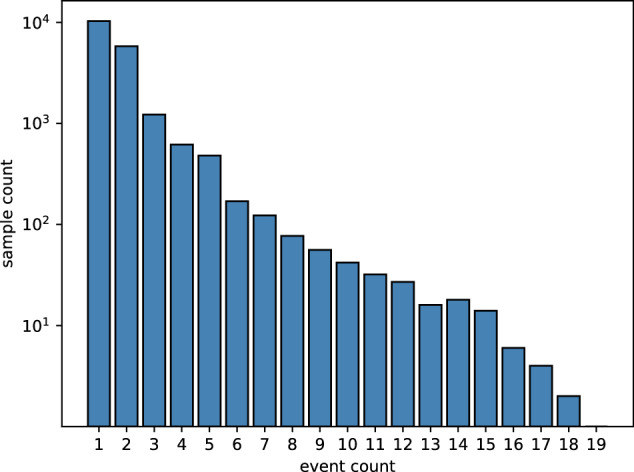
Distribution of the number of events within a single document.

**Fig. 7:**
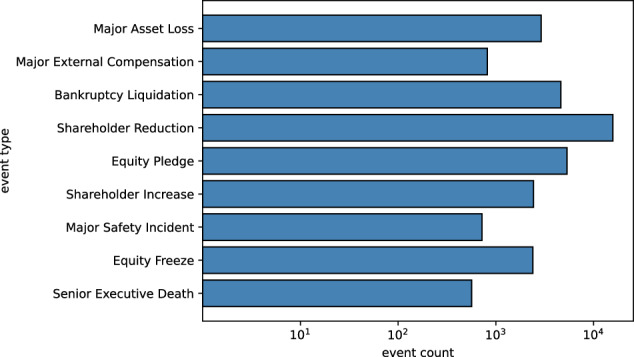
Comparison of the occurrence frequency of different event types across the entire dataset.

Figure 6 displays the distribution of the number of events within a single document, with the maximum number of events in a document being 19 and a substantial number of documents containing more than one event. Fig. 7 illustrates the distribution of data volume for different event types, with the most frequently mentioned event being the Shareholder Reduction, as each instance of this event is potentially mentioned multiple times within a single announcement. The least frequent event type is executive death, which is inherently rare.

**Fig. 8:**
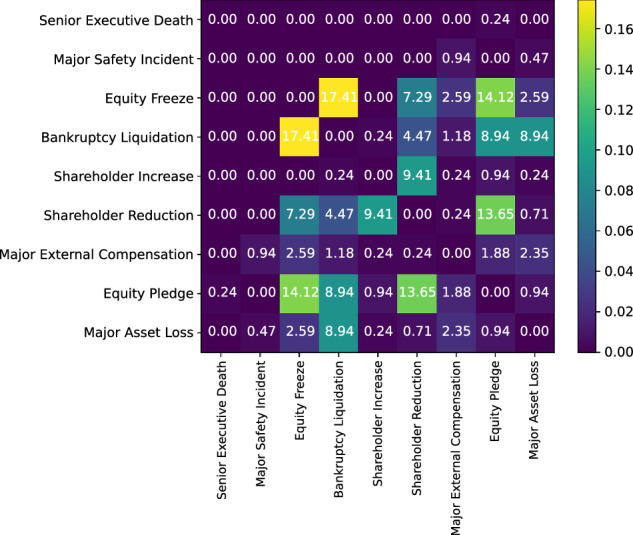
Statistical analysis of the co-occurrence patterns of various event types within a single announcement.

Figure 8 illustrates the co-occurrence ratios of different event types within the same document. It can be observed that the event of Equity Freeze frequently appears in the announcements of Bankruptcy Liquidation. Moreover, the events of Equity Pledge and Shareholder Reduction, along with Equity Freeze, often occur concurrently. These co-occurrence patterns are consistent with the objective laws of the financial market and can be utilized to enhance the effectiveness of financial event extraction methodologies.

## Data Records

The dataset DocFEE^30^ is available at Figshare (10.6084/m9.figshare.28632464) and OpenDataLab (https://opendatalab.com/tongzhou21/DocFEE). The dataset folder includes files that explain the event framework definitions and the specific annotated data, the detailed description available at “README.pdf”.

### Event Schema

The event types and their corresponding arguments are described in the file “schema.json” This file is stored in the form of a JSON dictionary, where the keys are event type labels and the values are lists of event arguments corresponding to each event type.

### Annotated Data

The annotated data includes two files for the training and validation “train.jsonl”, and test “test.jsonl” sets, all organized in the same format. Each line represents an annotation and is stored as a JSON dictionary containing the document content and all corresponding financial event dictionaries. Each event dictionary includes the event type name, all instances of event arguments, and the text segments in the document corresponding to those arguments. The format is shown in Table 5, and “sample.json” provides an example of the data.

**Table 5:** Format of each data record: Field, Label, Content.

Field	Type	Content
content	String	Chinese financial document
events	Array of JSON Objects	List of event information, including event type and corresponding event arguments. E.g. {"event_type”: “Shareholder Increase”, “increase_amount”: “5280621”, “increase_start_date”: “2019.07.11”, “shareholder_who_increased”: “Zibo Blue Sail Investment Co., Ltd.”}

## Technical Validation

In this section, we will assess and validate the credibility and effectiveness of the DocFEE dataset. We employ expert manual evaluation to directly assess the credibility, thereby substantiating the quality of the dataset annotations. Regarding the effectiveness of the dataset, an indirect assessment approach is utilized, which involves examining the enhancement in the performance of various algorithms when the dataset is used as a training set. We will now proceed to detail these two aspects.

### Credibility

The accuracy of manual verification at each step of the dataset construction pipeline is summarized in Table 6. The average cost of the entire annotation process is 0.002 USD per data entry, representing 0.4% of the cost associated with human-only annotation. The final dataset attains an annotation accuracy of 84.04%. In contrast, the direct application of GPT-3.5-turbo for annotation yielded a usability rate of 61.05%, and the annotation cost was also higher than our pipeline. This enhancement in performance underscores the efficacy of the collaborative financial document-level event extraction annotation pipeline. Additionally, the iterative optimization process involving humans in the loop offers the potential for continuous improvement in the dataset’s quality. To assess the reliability of our automatic annotation process, we calculated Cohen’s Kappa (*κ* = 0.78) on a randomly selected 500-sample subset, which was independently annotated by three instances of HAC-Ann. This substantial agreement indicates a high level of consistency in the automatic annotation process, thereby supporting the reliability and potential accuracy of our dataset.

**Table 6:** Comparison of the cost and effectiveness of different annotation methods.

Method	Price / 1k sample	Arguments F1
Qwen1.5-14B-Chat	0.05	46.66%
Qwen1.5-32B-Chat	0.09	49.66%
gpt-3.5-turbo-0125	5	61.05%
gpt-4o	50	59.60%
gpt-3.5-turbo-0125 w SG	14	79.79%
gpt-4o w SG	112	77.89%
human	500	100.00%
HAC-Ann (Ours)	2	84.04%

The F1 score for event arguments is derived from the comparative annotations of different annotation models on a manually annotated dataset.

### Effectiveness

The effectiveness of the annotated dataset DocFEE was validated through a supervised learning paradigm on subsets of our dataset with varying sizes, as detailed in Table 7. We aim to investigate whether the state-of-the-art document-level event extraction methods can benefit from being trained on our dataset. The baseline methods span various paradigms:

**Table 7:** The comparison of event argument extraction across various methods trained on datasets of different scales.

Method	4k			8k			18k		
	P	R	F	P	R	F	P	R	F
BERT_event_cls	0.8051	0.9814	0.8846	0.8183	0.9864	0.8945	0.8361	0.9851	0.9045
BART_QA	0.1069	0.1780	0.1336	0.1720	0.3068	0.2204	0.1982	0.3139	0.2430
BERT_Tagging	0.4387	0.2595	0.3261	0.4609	0.2746	0.3442	0.4336	0.3131	0.3636
LLM_SFT	0.6619	0.6791	0.6704	0.7213	0.7015	0.7112	0.7315	0.7186	0.7250

BERT_event_cls refers to the accuracy of determining the event type in a document using the BERT model.

**BERT_Tagging**^17^: This approach directly performs sequence tagging for target events. Each event argument is assigned its corresponding B (Begin) and I (Inside) sequence tag. Argument content from the dataset is mapped back to its position in the document, serving as the target label. Training and inference handle financial documents exceeding BERT’s predefined maximum token input length using a sliding window approach. After the model predicts all arguments in the document, these arguments from each chunk are then merged from adjacent events without identical event arguments.

**BART_QA**^31^: This two-stage method first classifies events within documents and then utilizes a reading comprehension-style question-answering approach for target event argument prediction. A sliding window approach is used to deal with long-form documents. We first employ BERT for multi-label classification to predict target event types. Each document segment is queried for all corresponding event arguments based on the known event types, outputting the start and end positions of corresponding arguments within the segment. The final merging process for event arguments is identical to BERT_Tagging.

**LLM_SFT:** We utilize Qwen1.5-7b-Chat for further finetuning due to its context window support up to 32k. This approach processes the entire document end-to-end based on an elaborate manual design prompt template, outputting all expected event types and arguments in a predefined JSON format^32,33^.

### Experimental Findings

The experimental results in Table 7 reveal that: The best performance of supervised training on the annotated dataset (LLM_SFT trained on 18k labeled data) outperforms the state-of-the-art commercial LLM, affirming the necessity of a well-annotated training set. Comparing the performance across training on 4k, 8k, and 18k labeled data, methods across different paradigms show progressive performance improvement with more training data, validating the effectiveness of the proposed large-scale, high-quality data annotation approach.

## Data Availability

The scripts utilized to crawl the raw annoucement, preprocess announcement, and auto-labeling pipeline are publicly available at the GitHub repository https://github.com/tongzhou21/DocFEE. Researchers working with the HAC-Ann to extend annotated datasets initially need to procure announcement documents through web scraping (spider.py). The candidate set for annotation is narrowed down through rule-based filtering (write_category_label.py) and local llm filtering local llm filtering (auto_label_qwen.py). Finally, a super-generative strategy (auto_label_openai.py) is employed for large-scale automatic annotation. During this period, manual annotation results can be reviewed to optimize the prompts (prompts/) further.
